# Vindication of the Medical Staff

**Published:** 1918-01-05

**Authors:** 


### AN INQUIRY AT LAMBETH INFIRMARY.

### Vindication of the Medical Staff.

LAMBETH Board of Guardians have held an inquiry, sitting in committee, into certain allegations which have been made regarding the medical and administrative control of their infirmary. The notice summoning the meeting had for its object to hold an inquiry into (*a*) the terms of the appointment of the medical staff of the infirmary, and the nature and amount of additional fees received by them; (*b*) the office accommodation of the clerical staff of the infirmary; and (*c*) any allegations respecting the "lack of supervision of the chief medical officer," and the "lack of co-ordination of the medical staff."

Mr. Frank Briant, J.P., L.C.C., presided over a large attendance of members, and a copy of the minutes was supplied to the representatives of the press at the last ordinary meeting of the Board. These stated that the Clerk submitted the following memorandum on sections (*a*) and (*b*):—

(*a*)—The permanent medical staff at the infirmary consists of Dr. Baly, medical officer since January 1911, salary, £400; Dr. Stebbing, first assistant medical officer since January 1909, salary, £450; Dr. Bowen (in the Navy), second assistant medical officer since May 1909, salary, £250; and Dr. Keats, assistant medical officer of workhouse (fourth assistant medical officer) since November 1909, salary, £7 7s. per week.

Apart from the temporary staff, it was noted that Dr. Baly is also medical officer of the Renfrew Road and Prince's Road Workhouses and the first four relief districts, for which he receives £150 per annum.

### THE DUTIES OF THE MEDICAL OFFICER.

The Clerk stated that the terms of appointment of the medical officer are sent out in a Special Order of the Local Government Board, dated August 25, 1873, as amended by the Special Order of September 13, 1911. The most important of these regulations are as follows:—

*Article* 42.—(2) To attend duly and punctually upon the paupers in the infirmary, according to the necessities of their cases, and to give the requisite directions as to their treatment, nursing, and diet, and the ventilation and the condition of the wards in which they are placed.

(3) To admit every pauper brought to the infirmary with the proper order, or transferred from the workhouse, and to examine his state on his admission, and to give the requisite directions as to his being placed in a ward appropriated to his class of case to which he belongs. Provided that, if in any case the medical officer is of opinion that the person sent in is not fit to be retained in the infirmary, he shall take a written report of the case to the Guardians at their next meeting, and take their directions thereon.

(4) To give the requisite directions as prescribed in Article 5 of this Order with respect to the transfer of paupers from the workhouse to the infirmary, and to make the written reports to the Guardians required by that Article, and Article 6 in the case of paupers who are transferred from the workhouse to the infirmary, or are sufficiently recovered to leave the infirmary.

(5) To report in writing to the Guardians any defect in the diet, drainage, furniture, ventilation, warmth, or other arrangements of the infirmary, or any excess in the number of the inmates, whether in the infirmary generally or in any particular ward, which he may deem to be detrimental to the health of the inmates, or calculated to retard their recovery.

(10) To give to the Guardians when required any reasonable information respecting the case of any pauper who is or has been under his care; to make any such written report relative to any sickness prevalent amongst the paupers in the infirmary as the Guardians or the Local Government Board may require of him; and to attend any meeting of the Guardians when requested by them to do so.

(11) To govern and control all the officers, assistants, and servants in the infirmary in conformity with the terms of this Order and the regulations prescribed by the Guardians, and from time to time to inform the visiting committee and the Guardians of the state of the infirmary in every department, and to report, when he deems necessary, in writing, to the Guardians any negligence or other misconduct on the part of any of the officers, assistants, or servants which shall come to his knowledge; and generally to observe and fulfil all lawful orders and directions of the Guardians suitable to his office.

The matters dealt with in the omitted clauses refer to detail or method.

### THE EXTRA FEES.

With regard to the appointment of Dr. Baly, the Clerk reported the conditions of appointment as settled by the Board on October 19, 1910.

The total fees received during the past year by the medical officer are as follows:—Lunacy fees (1917), £256 10s. 6d.; vaccination fees, £4 12s. 6d.; fees for examination of officers, £7 12s. 6d.

The Guardians do not interfere with any arrangement the medical officer may make with his assistants in connection with the allocation of any of the above fees. Assistant medical officers acting for the districts receive a fee of 10s. 6d. for each outdoor maternity case that they attend.

Dr. Baly was appointed to succeed Dr. Quarry, who resigned on August 31, 1910, having been given leave of absence, on account of sickness, in March of that year, from which date Dr. Baly had temporary charge of the infirmary. The conditions of the appointment, which (with the exception of £50 increase in the salary) were practically identical with those of Dr. Quarry, were settled in October, 1910, and in January 1911 Dr. Baly was appointed.

### NO ALLEGATIONS.

The Chairman drew attention to Clause (*c*) of the subject of their inquiry—any allegations respecting the "lack of supervision of the chief medical officer," and the "lack of co-ordination of the medical staff," and invited Guardians to submit any allegation they might wish to make on the points mentioned. No allegations, however, were put forward.

